# Supplementary material for: Myoglobin-derived iron causes wound enlargement and impaired regeneration in pressure injuries of muscle
Source: eLife. 2023 Jun 2;12:e85633. doi: 10.7554/eLife.85633 (PMC10238093; doi:10.7554/eLife.85633)
Supplement: Supplementary file 1. — (a) Injuries to the panniculus carnosus muscle from CTX and mPI have comparable diameters at day 3, but are significantly different at day 10. (b) Schematic showing differences in injury response and regeneration between cardiotoxin (CTX, acute injury) and muscle pressure injury (mPI, chronic wound). (c) External wound area in Myoglobin−/− and age- and sex-matched Myoglobin+/+ mice in the initial days following mPI, using 5 mm magnets. (d) Luminex measures of various cytokines, chemokines and growth factors between Myoglobin+/+ and Myoglobin−/− tissues, three days after mPI. (e) Treatment arms for 5-month-old mice with mPI. (f) Luminex measures of various cytokines, chemokines and growth factors between saline- and DFO-treated, three days after mPI. (g) Luminex measures of various cytokines, chemokines and growth factors between saline- and DFO-treated, ten days after mPI. (h) Specific pathogen free status of animal housing facility. [file elife-85633-supp1.docx]

Supplementary Material for

**“Myoglobin-derived iron causes wound enlargement and impaired regeneration in pressure injuries of muscle”**

N. Jannah M. Nasir, Hans Heemskerk, Julia Jenkins, N. Hidayah Hamadee, Ralph Bunte, and Lisa Tucker-Kellogg

| **Time-point** | **Cardiotoxin injury, diameter of dead muscle (mm)** | **Muscle pressure injury, diameter of dead muscle (mm)** | **p-value** |
| --- | --- | --- | --- |
| Day 3 | 8.44 ± 0.89 | 8.23 ± 0.98 | 0.5782 (ns) |
| Day 10 | 0.00 ± 0  (muscle has regenerated) | 5.75 ± 0.97 | < 0.0001 (****) |

**Supplementary File 1a:** Injuries to the panniculus carnosus muscle from CTX and mPI have comparable diameters at day 3, but are significantly different at day 10. Mean ± standard deviation. *n* = 3 mice. ns not significant, ****<0.0001 Statistical significance was computed by unpaired Student’s t test.

| **Wound healing milestones** | **Cardiotoxin (Acute injury)** | | | | **Pressure injury (Chronic wound)** | | | |
| --- | --- | --- | --- | --- | --- | --- | --- | --- |
|  | 3 Days | 10 Days | 40 Days | 90 Days | 3 Days | 10 Days | 40 Days | 90 Days |
| Immune cells pervade the wounded area | **🗸** |  |  |  | No | **🗸** |  |  |
| Dead tissue fully cleared | No | **🗸** |  |  | No | No | **🗸** |  |
| Wounded area revascularized | No | **🗸** |  |  | No | No | **🗸** |  |
| Immature myotubes have begun to form | No | **🗸** |  |  | No | No | **🗸** |  |
| Immature myotubes fill the wounded area | No | **🗸** |  |  | No | No | No | No |
| Mature muscle fibers fill the wounded area | No | No | **🗸** |  | No | No | No | No |

**Supplementary File 1b:** Schematic showing differences in injury response and regeneration between cardiotoxin (CTX, acute injury) and muscle pressure injury (mPI, chronic wound).

| **Time-point** | **External Wound Area** | |
| --- | --- | --- |
|  | ***Myoglobin^+^*^/+^** | ***Myoglobin*^−/−^** |
| Day 0 | 0.0 cm^2^ | 0.0 cm^2^ |
| Day 1 | 0.290 ± 0.069 cm^2^ | 0.224 ± 0.016 cm^2^ |
| Day 2 | 0.254 ± 0.050 cm^2^ | 0.165 ± 0.034 cm^2^ |
| Day 3 | 0.219 ± 0.0314 cm^2^ | 0.105 ± 0.083 cm^2^ |

**Supplementary File 1c:** External wound area in *Myoglobin*^−/−^ and age- and sex-matched *Myoglobin^+^*^/+^ mice in the initial days following mPI, using 5 mm magnets. Mean ± standard deviation.  *n* = 1 mouse each

| Analyte | Measurement | | p-value |
| --- | --- | --- | --- |
|  | *Myoglobin*^+/+^ | *Myoglobin*^−/−^ |  |
| CCL3 | 0.398 ± 0.312 pg/ml | 0.364 ± 0.096 pg/ml | 0.889 (ns) |
| CCL5 | 39.3 ± 3.13 pg/ml | 42.5 ± 10.1 pg/ml | 0.690 (ns) |
| CCL7 | 1.5786 ± 0.0340 pg/ml | 2.58 ± 0.512 pg/ml | 0.051 (ns) |
| CXCL12 | 296.62 ± 109.45 pg/ml | 150 ± 24.7 pg/ml | 0.138 (ns) |
| CXCL16 | 12.6 ± 2.85 pg/ml | 3.55 ± 0.54 pg/ml | 0.011 (*) |
| IL1b | 162.11 ± 164.06 pg/ml | 232 ± 144 pg/ml | 0.673 (ns) |
| IL4 | 21.616 ± 8.311 pg/ml | 21.0 ± 5.08 pg/ml | 0.937 (ns) |
| IL6 | 14.026 ± 1.591 pg/ml | 15.5 ± 1.54 pg/ml | 0.410 (ns) |
| IL10 | 4.5928 ± 0.0456 pg/ml | 4.66 ± 0.237 pg/ml | 0.722 (ns) |
| RAGE/AGER | 52.95 ± 47.448 pg/ml | 88.5 ± 23.2 pg/ml | 0.395 (ns) |
| uPAR | 262.47 ± 43.09 pg/ml | 485 ± 168 pg/ml | 0.144 (ns) |
| VEGF | 64.441 ± 3.711 pg/ml | 56.1 ± 19.3 pg/ml | 0.581 (ns) |
| PDGF-AA | 27.247 ± 6.276 pg/ml | 17.2 ± 7.48 pg/ml | 0.219 (ns) |
| PAI-1 | 22.5 ± 2.68 pg/ml | 36.9 ± 3.12 pg/ml | 0.008 (**) |
| IGF1 | 28.5 ± 3.44 pg/ml | 28.0 ± 4.97 pg/ml | 0.902 (ns) |
| Endoglin | 90.3 ± 82.4 pg/ml | 2406 ± 3355 pg/ml | 0.384 (ns) |

**Supplementary File 1d:** Luminex measures of various cytokines, chemokines and growth factors between *Myoglobin^+^*^/+^ and *Myoglobin*^−/−^ tissues, three days after mPI. Mean ± standard deviation. *n* = 6-7. Statistical significance was computed using a Student’s t-test with Bonferroni-Dunn correction.

| Treatment | Endpoint | # of mice |
| --- | --- | --- |
| Saline  control | 3 days | n = 7 |
|  | 7 days | n = 4 |
|  | 10 days | n = 7 |
|  | 40 days | n = 7 |
|  | 90 days | n = 5 |
| DFO | 3 days | n = 7 |
|  | 7 days | n = 4 |
|  | 10 days | n = 7 |
|  | 40 days | n = 7 |
|  | 90 days | n = 5 |

**Supplementary File 1e:** Treatment arms for 5-month-old mice with mPI. Both sexes were used. Mice were sex-matched and age-matched, and littermate controls were chosen when available.

| Analyte | Measurement | | p-value |
| --- | --- | --- | --- |
|  | Saline-treated | DFO-treated |  |
| CCL3 | 1680 ± 275 pg/ml | 1360 ± 539 pg/ml | 0.207 (ns) |
| CCL5 | 166 ± 102 pg/ml | 117 ± 47.0 pg/ml | 0.312 (ns) |
| CCL7 | 258 ± 260 pg/ml | 280 ± 276 pg/ml | 0.892 (ns) |
| CXCL12 | 3500 ± 3590 pg/ml | 3070 ± 4310 pg/ml | 0.854 (ns) |
| CXCL16 | 200 ± 45.6 pg/ml | 128 ± 39.0 pg/ml | 0.017 (*) |
| IL1b | 625 ± 662 pg/ml | 299 ± 177 pg/ml | 0.267 (ns) |
| IL4 | 44.1 ± 32.2 pg/ml | 26.9 ± 10.6 pg/ml | 0.003 (**) |
| IL6 | 95.6 ± 67.5 pg/ml | 77.4 ± 30.5 pg/ml | 0.558 (ns) |
| IL10 | 30.7 ± 27.8 pg/ml | 9.18 ± 3.97 pg/ml | 0.085 (ns) |
| RAGE/AGER | 128 ± 172 pg/ml | 0.00 pg/ml | 0.093 (ns) |
| uPAR | 12800 ± 9739 pg/ml | 6840 ± 3080 pg/ml | 0.179 (ns) |
| VEGF | 1060 ± 457 pg/ml | 981 ± 431 pg/ml | 0.761 (ns) |
| PDGF-AA | 209 ± 85.5 pg/ml | 165 ± 81.3 pg/ml | 0.378 (ns) |
| PAI-1 | 7850 ± 2140 pg/ml | 11400 ± 9550 pg/ml | 0.386 (ns) |
| IGF1 | 2540 ± 834 pg/ml | 2540 ± 1600 pg/ml | 0.996 (ns) |
| Endoglin | 41600 ± 27500 pg/ml | 15600 ± 14400 pg/ml | 0.063 (ns) |

**Supplementary File 1f:** Luminex measures of various cytokines, chemokines and growth factors between saline- and DFO-treated, three days after mPI. Mean ± standard deviation. *n* = 6-7. Statistical significance was computed using a Student’s t-test with Bonferroni-Dunn correction.

| Analyte | Measurement | | p-value |
| --- | --- | --- | --- |
|  | Saline-treated | DFO-treated |  |
| CCL3 | 1880 ± 1060 pg/ml | 1680 ± 855 pg/ml | 0.743 (ns) |
| CCL5 | 230 ± 143 pg/ml | 168 ± 67.8 pg/ml | 0.508 (ns) |
| CCL7 | 557 ± 479 pg/ml | 303 ± 228 pg/ml | 0.363 (ns) |
| CXCL12 | 8920 ± 9850 pg/ml | 3720 ± 3240 pg/ml | 0.251 (ns) |
| CXCL16 | 315 ± 181 pg/ml | 185 ± 50 pg/ml | 0.101 (ns) |
| IL1b | 726 ± 408 pg/ml | 569 ± 349 pg/ml | 0.507 (ns) |
| IL4 | 57.1 ± 13.8 pg/ml | 46.3 ± 18.9 pg/ml | 0.309 (ns) |
| IL6 | 317 ± 283 pg/ml | 160 ± 103 pg/ml | 0.236 (ns) |
| IL10 | 33.9 ± 25.7 pg/ml | 48.6 ± 42.3 pg/ml | 0.508 (ns) |
| RAGE/AGER | 436 ± 536 pg/ml | 268 ± 277 pg/ml | 0.913 (ns) |
| uPAR | 13400 ± 9250 pg/ml | 14100 ± 6060 pg/ml | 0.889 (ns) |
| VEGF | 365 ± 485 pg/ml | 2480 ± 2130 pg/ml | 0.324 (ns) |
| PDGF-AA | 172 ± 111 pg/ml | 213 ± 141 pg/ml | 0.602 (ns) |
| PAI-1 | 23500 ± 36300 pg/ml | 14900 ± 5750 pg/ml | 0.425 (ns) |
| IGF1 | 5670 ± 4800 pg/ml | 3120 ± 1440 pg/ml | 0.244 (ns) |
| Endoglin | 38800 ± 22400 pg/ml | 44800 ± 21100 pg/ml | 0.657 (ns) |

**Supplementary File 1g:** Luminex measures of various cytokines, chemokines and growth factors between saline- and DFO-treated, ten days after mPI. Mean ± standard deviation. *n* = 6-7. Statistical significance was computed using a Student’s t-test with Bonferroni-Dunn correction.

| **Health Report – Duke-NUS Vivarium** | | | | | | | | | |  |  |
| --- | --- | --- | --- | --- | --- | --- | --- | --- | --- | --- | --- |
| Sentinel Strain: ICR | Species: Mice | | | | Health Status: SPF | | | | |  |  |
| Room: SPF room |  |  |  |  |  |  |  |  |  |  |  |
| **Test Reference:** | **7341-2019** | **22559-2019** | **33873-2019** | **9872-2020** | | **24231-2020** | **33654-2020** | **11511-2-21** | **Test Laboratory** | | **Test Methods** |
| **Date tested:** | **6/3/2019** | **7/8/2019** | **6/12/2019** | **30/3/2020** | | **23/9/2020** | **18/12/2020** | **7/4/2021** |  | |  |
| **SEROLOGY (QUARTERLY)** |  |  |  |  | |  |  |  |  | |  |
| Ectromelia Virus (Mousepox) | 0/5 | 0/5 | 0/5 | 0/6 | | 0/6 | 0/6 | 0/6 | RADIL | | PCR/MFI |
| Epizootic diarrhoea of Infant Mice (EDIM) | 0/5 | 0/5 | 0/5 | 0/6 | | 0/6 | 0/6 | 0/6 | RADIL | | PCR/MFI |
| Lymphocytic Choriomeningitis Virus (LCMV) | 0/5 | 0/5 | 0/5 | 0/6 | | 0/6 | 0/6 | 0/6 | RADIL | | PCR/MFI |
| Mycoplasma pulmonis | 0/5 | 0/5 | 0/5 | 0/6 | | 0/6 | 0/6 | 0/6 | RADIL | | PCR/MFI |
| Mouse Hepatitis Virus (MHV) | 0/5 | 0/5 | 0/5 | 0/6 | | 0/6 | 0/6 | 0/6 | RADIL | | PCR/MFI |
| Murine norovirus (MNV) | 0/5 | 0/5 | 0/5 | 0/6 | | 0/6 | 0/6 | 0/6 | RADIL | | PCR/MFI |
| Mouse parvovirus (MPV) | 0/5 | 0/5 | 0/5 | 0/6 | | 0/6 | 0/6 | 0/6 | RADIL | | PCR/MFI |
| Minute Virus of Mice (MVM) | 0/5 | 0/5 | 0/5 | 0/6 | | 0/6 | 0/6 | 0/6 | RADIL | | PCR/MFI |
| Pneumonia virus of mice (PVM) | 0/5 | 0/5 | 0/5 | 0/6 | | 0/6 | 0/6 | 0/6 | RADIL | | PCR/MFI |
| Mouse Reovirus Type 3 | 0/5 | 0/5 | 0/5 | 0/6 | | 0/6 | 0/6 | 0/6 | RADIL | | PCR/MFI |
| Theiler's Murine Encephalomyelitis Virus | 0/5 | 0/5 | 0/5 | 0/6 | | 0/6 | 0/6 | 0/6 | RADIL | | PCR/MFI |
| Sendai Virus | 0/5 | 0/5 | 0/5 | 0/6 | | 0/6 | 0/6 | 0/6 | RADIL | | PCR/MFI |
| CAR bacillus | 0/5 | - | - | 0/6 | | - | - | 0/6 | RADIL | | PCR/MFI |
| Encephalitozoon cuniculi | 0/5 | - | - | 0/6 | | - | - | 0/6 | RADIL | | PCR/MFI |
| MAD1 | 0/5 | - | - | 0/6 | | - | - | 0/6 | RADIL | | PCR/MFI |
| MAD2 | 0/5 | - | - | 0/6 | | - | - | 0/6 | RADIL | | PCR/MFI |
| Polyoma Virus | 0/5 | - | - | 0/6 | | - | - | 0/6 | RADIL | | PCR/MFI |
| Clostridium piliforme | 0/5 | - | - | 0/6 | | - | - | 0/6 | RADIL | | PCR/MFI |
| Mouse Cytomegalovirus | 0/5 | - | - | 0/6 | | - | - | 0/6 | RADIL | | PCR/MFI |
| **BACTERIOLOGY (ANNUALLY)** | **6/3/2019** |  |  | **30/3/2020** | |  |  | **7/4/2020** |  | |  |
| *Citrobacter rodentium* | 0/5 | - | - | 0/6 | | - | - | 0/6 | RADIL | | PCR |
| *Klebsiella oxytoca* | 0/5 | - | - | 0/6 | | - | - | 0/6 | RADIL | | PCR |
| *Klebsiella pneumoniae* | 0/5 | - | - | 0/6 | | - | - | 0/6 | RADIL | | PCR |
| *Proteus mirabilis* | 0/5 | - | - | 0/6 | | - | - | 0/6 | RADIL | | PCR |
| *Pseudomonas aeruginosa* | 0/5 | - | - | 0/6 | | - | - | 0/6 | RADIL | | PCR |
| *Salmonella sp.* | 0/5 | - | - | 0/6 | | - | - | 0/6 | RADIL | | PCR |
| *Bordetella bronchiseptica* | 0/5 | - | - | 0/6 | | - | - | 0/6 | RADIL | | PCR |
| *Corynebacterium kutscheri/bovis* | 0/5 | - | - | 0/6 | | - | - | 0/6 | RADIL | | PCR |
| *Pasteurella pneumotropica* | 0/5 | - | - | 0/6 | | - | - | 0/6 | RADIL | | PCR |
| *Pasteurella multocida* | 0/5 | - | - | 0/6 | | - | - | 0/6 | RADIL | | PCR |
| *Staphylococcus aureus* | 0/5 | - | - | 0/6 | | - | - | 0/6 | RADIL | | PCR |
| *Streptococcus pneumoniae* | 0/5 | - | - | 0/6 | | - | - | 0/6 | RADIL | | PCR |
| *Streptococcus sp. Beta hemolytic* | 0/5 | - | - | 0/6 | | - | - | 0/6 | RADIL | | PCR |
| *Streptobacillus moniliformis* | 0/5 | - | - | 0/6 | | - | - | 0/6 | RADIL | | PCR |
| *Helicobacter ganmani* | 0/5 | - |  | 0/6 | | - |  | 0/6 |  | |  |
| *Helicobacter hepaticus* | 0/5 | - | - | 0/6 | | - | - | 0/6 | RADIL | | PCR |
| *Helicobacter typhlonius* | 0/5 | - | - | 0/6 | | - | - | 0/6 | RADIL | | PCR |
| *Helicobacter mastomyrinus* | 0/5 | - | - | 0/6 | | - | - | 0/6 | RADIL | | PCR |
| *Helicobacter sp., H.bilis, H.rodentium, H.mastomyrinus* | 0/5 | - | - | 0/6 | | - | - | 0/6 | RADIL | | PCR |
| **PARASITOLOGY (QUARTERLY)** | **6/3/2019** | **15/8/2019** | **15/12/2019** | **30/3/2020** | | **28/9/2020** | **21/12/2020** | **7/4/2020** |  | |  |
| Ectoparasites (e.g., fleas, fur mites, lice) | 0/5 | 0/5 | 0/5 | 0/6 | | 0/6 | 0/6 | 0/6 | RADIL/SGH | | PCR/ LM |
| Endoparasites (pinworms, tapeworms, roundworms) | 0/5 | 0/5 | 0/5 | 0/6 | | 0/6 | 0/6 | 0/6 | RADIL/SGH | | PCR/LM |

Legends:

RADIL – Research Animal Diagnostic Laboratory

SGH – Singapore General Hospital (SGH Department of Pathology)

MFI – Multiplex Fluorescent Immunoassay

LM – Light Microscopy

**Supplementary File 1h: Specific pathogen free status of animal housing facility.** The SingHealth animal facility employs the sentinel method for surveillance of pathogens. Sentinel mice (ICR strain) are housed on soiled bedding removed from cages of other non-sentinel rodents in the population to be sampled. Sentinels are housed in individual ventilated cages like all other rodents. They are housed in the colony for at least 3 months before being tested. The table above details the specific pathogen tested for and the mode and frequency of testing. 0/6 indicates that zero out of six mice tested positive for that pathogen.
